# Supplementary material for: Development of a computer-aided design software for the quantitative evaluation of aesthetic damage
Source: PLoS One. 2019 Dec 18;14(12):e0226322. doi: 10.1371/journal.pone.0226322 (PMC6919621; doi:10.1371/journal.pone.0226322)
Supplement: S1 Code site — 1. (ZIP) [file pone.0226322.s001.zip › [EESC_jr][Projeto]Relatorio_07_21/reports/templates_zip/gentelella-master/vendors/jszip/index.html]

---
title: JSZip
layout: default
section: main
---

JSZip is a javascript library for creating, reading and editing .zip files, with a
lovely and simple API.

**Current version** : v2.6.0

**License** : JSZip is dual-licensed. You may use it under the
MIT license *or* the GPLv3 license. See
LICENSE.markdown.

### Example

var zip = new JSZip();
zip.file("Hello.txt", "Hello World\n");
var img = zip.folder("images");
img.file("smile.gif", imgData, {base64: true});
var content = zip.generate({type:"blob"});
// see FileSaver.js
saveAs(content, "example.zip");
Run!


This browser doesn't support blobs, this demo won't work :(
See here for more info.

### Installation

**With npm** : `npm install jszip`

**With bower** : `bower install Stuk/jszip`

**With component** : `component install Stuk/jszip`

**Manually** : download JSZip
and include the file `dist/jszip.js` or `dist/jszip.min.js`

  

Installed ? Great ! You can now check our
guides and examples !

### Support

| Opera | Firefox | Safari | Chrome | Internet Explorer | Node.js |
| --- | --- | --- | --- | --- | --- |
| Yes | Yes | Yes | Yes | Yes | Yes |
| Tested with the latest version | Tested with 3.0 / 3.6 / latest version | Tested with the latest version | Tested with the latest version | Tested with IE 6 / 7 / 8 / 9 / 10 | Tested with node.js 0.8 and 0.10 |

### Getting help

Having trouble ? We'd like to help !

- Try the FAQ, it has
  answers to common questions.
- If you're looking for informations about a specific method, try the
  documentation.
- Check the
  examples.
- Report bugs in our
  Bug tracker.

### Test status

Travis build :


Saucelabs build :


Live tests :
:   See for yourself !
